# Stability analysis of elastomeric impression materials after antimicrobial disinfection

**DOI:** 10.6026/9732063002001070

**Published:** 2024-09-30

**Authors:** Santosh T., Pushkar Gupta, Omar Basheer Altaher Mohammed, Aditi M Paranjpe, Harsh Chansoria, Abhishek Pathak, Dayanand Huddar, Sanchari Bhowmick

**Affiliations:** 1 Department of Oral Pathology & Microbiology, Vananchal Dental College & Hospital, Garhwa, Jharkhand, India; 2 Department of Prosthodontics and Crown & Bridge, Hitkarini Dental College and Hospital, Jabalpur, M.P, India; 3 Department of Oral and Maxillofacial Surgery, Sebha Dental Faculty, Sebha University, Libya; 4 Department of Prosthodontics and Crown and Bridge, Government College of Dentistry, Indore, Madhya Pradesh, India; 5 Department of Prosthodontics, Bharati Vidyapeeth Dental College and Hospital, Sangli, Maharashtra, India; 6 Intern, Kalinga Institute of Dental Sciences, KIIT Deemed to be University, Bhubaneswar, Odisha, India

**Keywords:** Disinfection, dimensional changes, elastomeric impression, sterilisation

## Abstract

Impression materials used for replication of dental structures frequently come into touch with blood and saliva during the impression operation. Hence, disinfection of impression mistrials is needed. Therefore, it is of interest to evaluate the effects of three different disinfectants on the antibacterial activity and dimensional stability of the elastomeric impression material. According to American Dental Association (ADA) specification number 19 compliant, a stainless steel master die was constructed. Using vinyl polysiloxanes (VPS) impression medium, thirty samples were produced on this die in accordance with the manufacturer's instructions. To finish the disinfection process, thirty samples were randomly assigned to each of the three groups: Group I consisted of diluted water (control group), Group II consisted of 5.25 percent sodium hypochlorite (NaOCl), and Group III consisted of ozone gas and Group IV - UV radiation. The dimensional stability was evaluated using a stereomicroscope with a 20x magnification and image research software. The antibacterial efficacy of each disinfectant was assessed. In the VPS impression material, the control group exhibited the greatest number of dimensional changes, with ozone gas and 5.25% NaOCl showing the least amount of dimensional changes. The groups were found to differ statistically significantly from one another. The results of the investigation indicate that the VPS elastomeric impression showed very minor dimensional changes when submerged in different disinfectants. In a clinical setting, samples cleaned with 5.25% hypochlorite can be kept for a long time because the consequent dimensional changes are minimal. Ozone gas, UV radiation and sodium hypochlorite showed a reduction in the amount of bacteria.

## Background:

Dentists routinely create impressions, and this process calls for selecting the appropriate instruments and supplies. Casts are made from the impressions once they are created, and these are used to make a range of appliances, such as study models or dies [1]. Impressions are required for certain dental procedures in order to produce accurate castings of the oral anatomy. During the imprint procedure, blood and saliva are regularly in contact with impression materials. This raises the possibility of infection with infectious diseases such as AIDS, herpes, hepatitis or tuberculosis. Impressions must always be cleaned since dental lab workers, oral hygienists, and dentists are often in contact with infectious diseases [2]. 1998 FDI regulations state that all impression materials need to be sanitised before being submitted to a laboratory [3]. Polyethers, some additional silicone compounds, and both reversible and irreversible hydrocolloids are more hydrophilic than other types of imprints. Elastomeric imprint materials are commonly used because of their favourable physical characteristics. Vinyl polyether silicones (VPES) are a new class of elastomeric impression materials with improved mechanical and flow properties. Polyethers (PE) and VPS impression materials are used to make final impressions for patients who are edentulous [4]. Owing to its low distortion and exceptional dimensional precision, poly-vinyl-siloxane is a commonly used silicone elastomeric imprint medium [2]. Disinfectants should not compromise the dimensional precision of the impression material; instead, their use should be commensurate with the effectiveness of antimicrobial agents. It is advised to clean impression materials using a range of disinfectants, such as sodium hypochlorite, phenol, iodophor, and glutaraldehyde [2]. One often used method is chemical disinfection, which includes dipping or spraying a chemical solution into the impression's surface. Dimensional stability must be demonstrated by the material both throughout the cleaning procedure and in storage until the cast is poured. As a result, it is crucial to keep the dimensional variations of the impression material within the allowed range of 0-0.15% [1]. To lessen the negative impact of chemical agents on the different material properties of dental impressions, researchers are investigating substitute disinfection methods such as ozone gas, ethylene oxide gas, microwave irradiation, UV radiation, and ozonated water [5, 6]. The precision of the finished product may be compromised if that chemical is used by spray or immersion because of its hydrophilic properties, which may cause imprints to be distorted and dental casts to be, produced [5]. Ozone is a gas composed of three oxygen atoms, whereas the oxygen we breathe is composed of two oxygen atoms. Because of its extreme instability and strong reactivity, ozone is a powerful steriliser. In addition, ozone is a potent oxidant that can harm intracellular enzymes, cell membranes, and even the DNA of microbes [7]. When disinfecting silicone impression materials, ozone water has been suggested as a clinical equivalent for 5.25% NaOCl and 2% glutaraldehyde [8]. Certain hypotheses suggest that disinfectants may alter the dimensional accuracy of impression materials [2]. Therefore, it is of interest to evaluate the effects of three different disinfectants on the antibacterial activity and dimensional stability of the elastomeric impression material.

## Materials and Method:

The current study was carried out by the Department of Prosthodontics and the Department of Oral Pathology. An American Dental Association (ADA) specification number 19 compliant stainless steel master die was constructed. Using vinyl polysiloxanes (VPS) impression medium, thirty samples were produced on this die in accordance with the manufacturer's instructions. To finish the disinfection process, thirty samples were randomly assigned to each of the three groups: Group I consisted of diluted water (control group), Group II consisted of 5.25 percent sodium hypochlorite (NaOCl), Group III consisted of ozone gas and Group IV: UV radiation. The mould consisted of a base with three horizontal and two vertical lines perpendicularly carved into it, each measuring 0.050 mm in width. Following the loading of the elastomers, pressure was applied using a base-mounted, precisely-positioned steel ring with an internal diameter of 3.8 mm and a perforated steel plate. First, light body material was immediately injected into the platform to place the metal ring on the base of the mould. The tray material was then blended and loaded in compliance with the manufacturer's instructions. Extra material was eliminated by perforation. The specimens were submerged in a water bath with a thermostat set to 37°C in order to simulate oral circumstances. Thirty specimens in all, ten for each set of materials, were made. The distance (0.005 mm) between the horizontal line's inner profiles was measured under a microscope both before and after each disinfectant application after the sample was created. Dimensional change % = (A-B)/A 100 was the formula used to determine the percentage of dimensional change. "A" stands for the distance between the inner profile of the horizontal line before disinfection, and "B" for the distance after disinfection. The dimensional stability was evaluated using a stereomicroscope with a 20x magnification and image research software. Thirty impressions of the patient's jaw were taken, and three distinct disinfectants were used to test the antibacterial efficiency of the impressions both before and after. Before and after disinfection, swabs from the molar region of each elastomeric imprint were taken, and they were then cultured for 24 hours at 37°C in nutritional agar media. The microbial colony count was carried out using a colony counter following a 24-hour period. Using the ANOVA test with p<0.05, the collected data was statistically assessed using SPSS software version 22.0.

## Results:

In the VPS impression material, the control group exhibited the greatest number of dimensional changes, with ozone gas, UV radiation and 5.25% NaOCl showing the least amount of dimensional changes. ANOVA showed that, with a p-value of less than 0.001, there were statistically significant differences between the groups (Table 1). Table 2 shows that the bacterial count significantly decreased in groups II, III and IV, although it decreased the least in the control group (Group I). There was a statistically considerable variation before and after disinfection.

## Discussion:

Dental imprints are used to make a negative depiction of the teeth and hard and soft mouth tissues found in the human dentition [5]. Accurate reproduction of the surface details of the oral structure is necessary after the impression has been created. Impression materials need to be cleaned in order to avoid cross-infection [2]. Immersion and spraying are the two main methods used to disinfect imprints. The spray method undercuts the antimicrobial agent and does not fully expose the contaminated area, whereas the immersion method covers all surfaces but is not ideal [9]. Elastomers are dimensionally unstable when used as impression resources because of partial elastic revival after deformation, temperature changes, or the discharge of chemical reaction by products during polymerization shrinkage [1]. The results of the investigation indicate that the VPS elastomeric impression showed very minor dimensional changes when submerged in different disinfectants. A variety of measurement techniques are used to determine the changes in dimensions that occur during disinfection. In some research, travelling microscopes are employed. With stone castings or impressions, dimensional stability and accuracy could be measured directly [4, 10]. Following immersion of vinyl polysiloxanes (VPS) and poly-ethers (PE) in two different disinfectants (a 5.25% sodium hypochlorite (NaOCl) group and a 2% glutaraldehyde (GA) group), Almuraikhi's research showed that the materials showed minimal changes in their dimensions [1]. Using the autoclave, chemical and microwave treatments as disinfectants, Kamble *et al.* evaluated the dimensional accuracy of elastomeric imprint materials. They concluded that the impression material has minor dimensional changes as a result of all disinfection methods. They asserted that chemical disinfection causes less dimensional changes than the autoclave and microwave procedures [2]. Vinyl siloxanether (VSE) and polyvinyl siloxane (PVS), two elastomeric impression materials, were disinfected by chemical immersion and microwave irradiation. Mohd *et al.* evaluated and contrasted these materials' dimensional stability. They concluded that VSE demonstrated higher dimensional stability than PVS under both chemical immersion and microwave irradiation. Microwave irradiation using regular microwave ovens can be used as a therapeutic substitute for other disinfection techniques [11]. Wezgowiec *et al.* evaluated the efficacy of UVC radiation, gaseous ozone, and commercial liquid chemicals for the disinfection of silicone dental impressions. They concluded that even while all of the treatments that were tested worked, each disinfectant still needed to be assessed separately [5]. After removing the Type IV gypsum casts from sanitised elastomeric impression materials, Pal *et al.* evaluated the casts' dimensional stability and surface quality. They concluded that all three disinfectants produced complete disinfection and that none of the disinfectants had an impact on the replication of surface details [12]. Karaman *et al.* assessed how long the application of sodium hypochlorite and a quaternary ammonium-based disinfection solution would roughen the surface of an elastomeric imprint material. They concluded that long-term application of the sodium hypochlorite disinfectant at 1% and 5% concentrations greatly increased the surface roughness of the light body elastomeric imprint material [13]. Ozone gas and sodium hypochlorite both demonstrated strong antibacterial effects in the current study, with the control group experiencing the least.

Nagi *et al.* evaluated and compared the disinfection effectiveness of a commercially available herbal formulation (HiOra®) with 1% sodium hypochlorite and 0.2% chlorhexidine digluconate solution on dental impressions made with condensation silicone. They concluded that the three disinfectants tested exhibited comparable antimicrobial efficacy against Streptococcus and Staphylococcus species, and that herbal mouthwash was equally effective as sodium hypochlorite and chlorhexidine at sanitising impressions made from condensation silicone [14]. According to Trivedi *et al.*, there is a mean percentage decrease in colony count following a three-minute immersion of *P. aeruginosa*, *S. aureus* and *C. albicans* in aloe vera and a three-minute disinfection spray. Complete destruction of every germ cell after a 7-minute immersion and spray cleaning [6]. Ahirwar *et al.* evaluated the effectiveness of spray disinfectants against oral germs on an irreversible hydrocolloid imprint material called alginate. They came to the conclusion that alginate imprints can be successfully disinfected with spray disinfectants that contain 2% glutaraldehyde and 0.5% sodium hypochlorite [9]. After immersion disinfection with two distinct disinfectants at three different time intervals, Soganci *et al.* measure and compare the dimensional changes of vinyl polyether siloxane impression materials and polyether impression materials. They came to the conclusion that both impression materials' dimensional accuracy and stability were outstanding and comparable [4]. Rathod *et al.* evaluated the antibacterial properties of a prepared herbal solution on dental impressions using irreversible hydrocolloid. This study is an ex vivo comparative analysis. They concluded that it works well to use a herbal disinfectant solution to stop germs from growing on imprints that contain irreversible hydrocolloid [15]. Among the drawbacks of this study include; in vitro design and the fact that the imprints created and eliminated differed from those created in clinical settings. To validate the results, more research is needed.

## Conclusion:

Data indicate that the VPS elastomeric impression showed very minor dimensional changes when submerged in different disinfectants. In a clinical setting, samples cleaned with 5.25% hypochlorite can be kept for a long time because the consequent dimensional changes are minimal. Antibacterial activity was highest in Sodium hypochlorites followed by and highest in the ozone gas and radiation group and it was lowest in control group.

## Source of funding:

Self

## Figures and Tables

**Table 1:** Analysing the average dimensional stability both prior to and during disinfection

**Disinfectant group**	**Before (mean ± SD)**	**After (mean ±SD)**	**Dimensional changes**	**F value**	**p**
I.Control group (Distilled water)	0.14±0.02	0.83 ± 0.12	0.78 ±0.09		
II. Sodium hypochlorite	0.14± 0.06	0.62±0.15	0.51±0.07	7.326	0
III. Ozone gas	0.13 ±0.04	0.53±0.16	0.44±0.13		
IV. UV radiation	0.12 ±0.01	0.56±0.19	0.41±0.12		

**Table 2:** Total bacterial count (CFU/ml), before and after disinfection

**Disinfectant group**	**Before (mean)**	**After (mean)**	**p**
I.Control group (Distilled water)	8.78x10^5^	6.52x10^5^	0
II. Sodium hypochlorite	8.81x10^5^	1.31x10^4^	
III. Ozone gas	8.77x10^5^	1.59x10^4^	
IV. UV radiation	8.72x10^5^	1.62x10^4^	
F value	0.241	171.018	
